# The impact of time-of-day reperfusion on remote ischemic conditioning in ST-elevation myocardial infarction: a RIC-STEMI substudy

**DOI:** 10.1007/s00380-023-02247-8

**Published:** 2023-03-17

**Authors:** Carla Marques Pires, Diana Lamas, António Gaspar, André P. Lourenço, Nuno Antunes, Jorge Marques, Adelino F. Leite-Moreira

**Affiliations:** 1 Department of Cardiology, Braga Hospital, Braga, Portugal; 2 Minho University, Braga, Portugal; 3 Department of Surgery and Physiology, UnIC@RISE, Faculty of Medicine of the University of Porto, Porto, Portugal

**Keywords:** ST-elevation myocardial infarction, Remote ischemic conditioning, Heart failure, Cardiac death, Time-of-day

## Abstract

Daytime variation affects the tolerance of cardiomyocytes to ischemia–reperfusion injury (IRI). This study aims to evaluate the impact of time-of-day reperfusion on clinical outcomes of remote ischemic conditioning (RIC) as an adjuvant to primary percutaneous coronary intervention(PPCI) in ST-elevation myocardial infarction(STEMI) patients. A post-hoc analysis of a prospective, single-center parallel 1:1 randomized trial (RIC-STEMI) was performed. This analysis included 448 STEMI patients previously randomized to either PPCI alone (PPCI group) (*n* = 217) or RIC as an adjuvant to PPCI (RIC + PPCI group) (*n* = 231). Moreover, the sample was divided according to the time of PPCI: night-morning (22 h-11h59min) (*n* = 216) or afternoon (12 h-21h59min) (*n* = 232) groups. The primary follow-up endpoint was a composite of cardiac death and hospitalization due to heart failure. There were no significant differences in the clinical characteristics and the follow-up outcomes between groups. The afternoon period (HR = 0.474; 95% CI 0.230–0.977; *p* = 0.043) and RIC (HR = 0.423; 95% CI 0.195–0.917; *p* = 0.029) were independent predictors of the primary follow-up endpoint. An univariate analysis showed a lower frequency of primary follow-up endpoint, just in the afternoon period (10.3%vs0.9%; *p* = 0.002), in the RIC + PPCI group. A multivariate analysis revealed that RIC was an independent predictor of the primary follow-up endpoint in the afternoon group (HR = 0.098; 95% CI 0.012–0.785; *p* = 0.029), but not in the night-morning group. In addition, the afternoon period was not an independent predictor of the primary follow-up endpoint when the multivariate analysis was performed in the PPCI group. In conclusion, this study showed an important cardioprotective effect of RIC, namely in the afternoon period, suggesting that the afternoon period enhances the cardioprotection induced by RIC.

**Supplementary Information:**

The online version contains supplementary material available at 10.1007/s00380-023-02247-8.

## Introduction

Ischemic heart disease, mainly myocardial infarction (MI), remains a leading cause of death worldwide [1]. Nowadays, reperfusion strategies promptly restore blood flow and allow the reduction of infarct size and mortality [2–4]. However, abrupt reperfusion can, paradoxically, cause additional damage, called ischemia–reperfusion injury (IRI), which may be responsible for up to 50% of final infarct size and contribute to heart failure (HF) development, partially compromising the beneficial effect of reperfusion [5, 6].

Remote ischemic conditioning (RIC) is a cardioprotective strategy, in which brief cycles of non-lethal ischemia and reperfusion are applied to a distant organ before, during or after a long period of myocardial ischemia [7]. The rational of RIC has been extensively reviewed and established in basic research but the results of the translational investigation are controversial and largely disappointing.

Sloth et al. (2014), enrolled 251 ST-elevation myocardial infarction (STEMI) patients and revealed a 51% decrease in all-cause mortality, non-fatal MI, stroke and HF in patients who underwent RIC [8]. White et al. (2015), included 197 STEMI patients and showed a 27% reduction in infarct size and a 19% reduction of myocardial edema, assessed by cardiac magnetic resonance (CMR), in patients who underwent RIC [9]. Eitel et al. (2015), in the LIPSIA CONDITIONING study involving 696 STEMI patients, revealed a decrease in infarct size in patients who underwent combined intrahospital RIC and post-conditioning when compared with conventional primary percutaneous coronary intervention (PPCI) group [10]. Gaspar et al. [11], in a prospective single-center randomized trial with 448 STEMI patients, showed improvement in outcomes (cardiac death or HF hospitalization) in patients undergoing RIC as an adjunct to PPCI, over a mean follow-up period of 2.1 years [11]. Nevertheless, Hausenloy et al. [12], 13), in the largest prospective multicenter study (CONDI2/ERICPPCI) with 5401 patients, did not show evidence that RIC reduced cardiac death, HF hospitalization, improved left ventricle ejection fraction (EF) [12] or reduced infarct size at six months by CMR [13].

Therefore, it remains unclear whether RIC, restricted to patients with higher risk and greater susceptibility, could be cardioprotective.

Cardiovascular diseases show diurnal variation, with a higher incidence of STEMI in the morning. Recently, Montaigne et al. (2018), revealed the influence of the daytime variation on tolerance to IRI in patients undergoing aortic valve replacement surgery. This study concluded that surgeries performed in the afternoon were associated with better clinical outcomes compared to those carried out in the morning [14].

Up till now, the influence of daytime variation in IRI and in clinical results of RIC has never been raised. Therefore, our aim was to assess whether RIC, as an adjuvant to PPCI, performed in STEMI patients in the afternoon period had different clinical results.

## Methods

### Study design

This study consists of a post-hoc analysis of the RIC-STEMI study (NCT02313961) [11], a single center, open label, parallel 1:1 randomized controlled trial.

This trial aimed to assess the superiority of RIC (3 cycles of inflation and deflation of a left lower limb cuff, for 5 min each) over PPCI in all-comer patients presenting with STEMI between March 2013 and December 2015. Eligible patients were at least 18 years old and were admitted to the emergency department of Braga Hospital with putative STEMI [15]. Exclusion criteria were cardiogenic shock, defined by a systolic blood pressure lower than 90 mmHg and evidence of tissue hypoperfusion; post-cardiac arrest status; the need for mechanical ventilation; known peripheral arterial disease; evidence of lower limb ischemia and recent MI (within the last 30 days).

To perform this study the authors consulted the database of RIC-STEMI study. This analysis included 448 STEMI patients previously randomized to either PPCI alone (PPCI group) (*n* = 217) or RIC as an adjuvant to PPCI (RIC + PPCI group) (*n* = 231). To assess the effect of daytime variation on RIC clinical results, the sample was divided according to the time of PPCI in night-morning (22 h–11 h59 min) or afternoon (12 h–21 h59 min) periods.

**Fig. 1:**
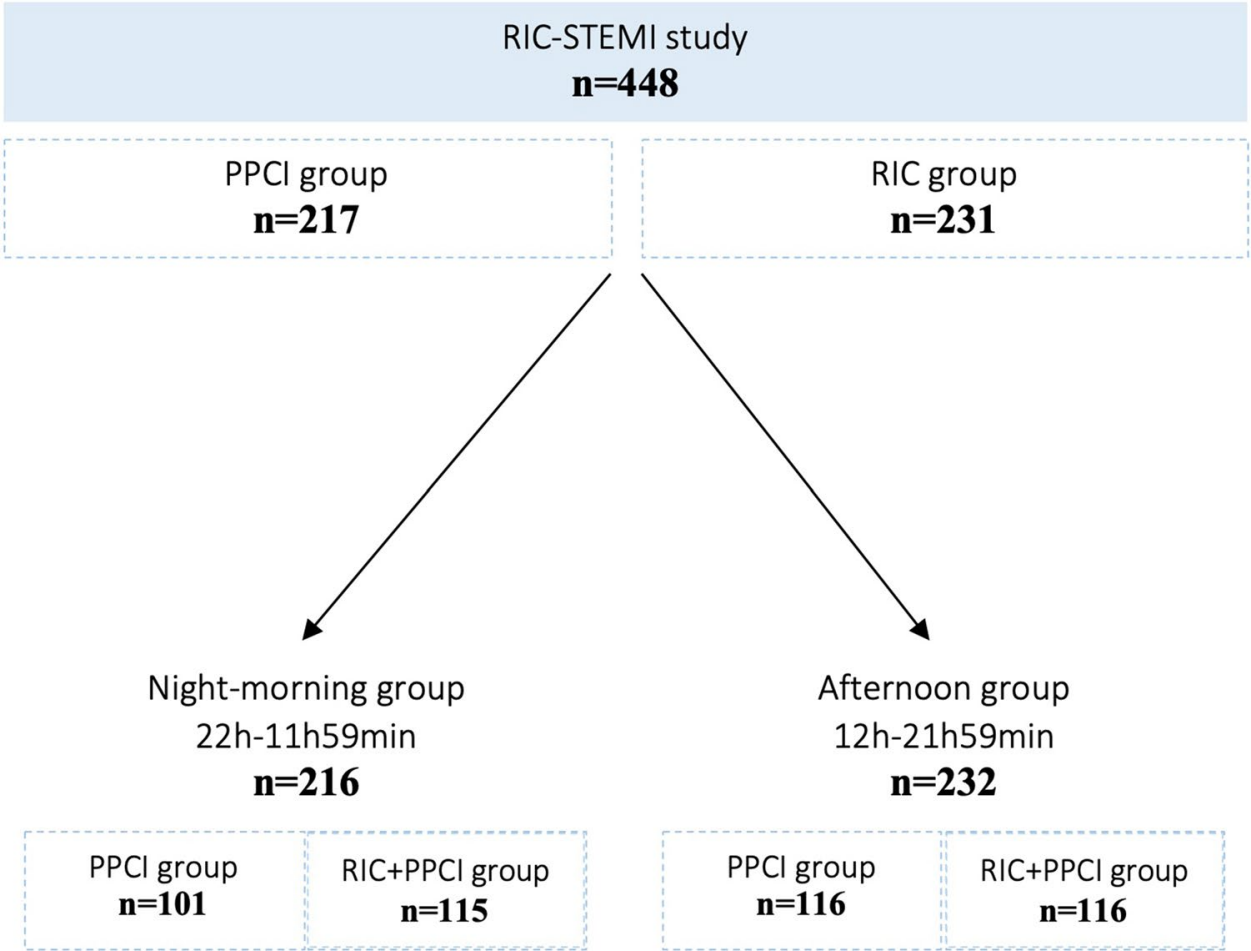
Study flow diagram

The cardioprotective effect of RIC was compared within isolated PPCI, according to the period of the day in which it was performed. Figure 1 shows the study flow diagram.

The admission echocardiography was performed in the first 24 h of hospitalization. The median month to measure the follow-up ejection fraction was 10 (IQR 1.8) months.

### Study endpoints

The primary follow-up endpoint was a composite of cardiac mortality and hospitalization due to HF.

The minimum predefined follow-up time was 12 months.

Secondary endpoints were follow-up EF (estimated by Simpson’s biplane method), major adverse cardiovascular and cerebrovascular events on follow-up (MACCE), hospitalization due to HF, all-cause mortality, and cardiac mortality.

Hospitalization due to HF included readmissions due to acute or chronically decompensated HF, planned implantation of cardioverter-defibrillator (ICD) or cardiac resynchronization therapy device (CRT).

Cardiac death was defined as natural death due to cardiac causes and required evidence of acutely decompensated HF or sudden cardiac arrest. Patients found dead at home were not considered.

MACCE consisted of the composite of total death, MI, stroke (persistent neurological deficit) and target vessel revascularization.

### Ethical procedures

This study was approved by Braga Hospital Ethical Committee and met the criteria established by the Declaration of Helsinki 1964 as revised in 2013 and the International Conference of Harmonisation Guidelines for Good Clinical Practice. All patients enrolled gave witness oral consent and written informed consent after clinical stabilization.

### Statistical analysis

Statistical analysis was performed using IBM^®^ SPSS^®^, version 27.

The normality of the distribution of continuous variables was evaluated through the Shapiro–Wilk’s test and histogram analysis. Continuous variables were described by median (Mdn) and interquartile range (IQ) since normality was not found in any of them. Categorical variables were described by absolute and relative frequencies (%).

The Mann–Whitney test was performed to compare continuous variables. The Chi-square test was used to compare categorical variables, although if the percentage of cells with < 5 expected counts were greater than 20%, Fisher’s exact test was preferred. Results with statistical significance were considered whenever the *p* value < 0.05.

If two variables were dichotomous, Phi (φ) was presented, as measured of effect size, considering the small, medium, or large effect for absolute values close to 0.10, 0.30 and 0.50, respectively.

Cox’s proportional hazards model with forward’s method was employed to evaluate the impact of RIC on the primary follow-up endpoint according to the period of the day in which PPCI was performed. Results are reported as hazard ratio (HR) and with a 95% of confidence interval (CI).

## Results

The RIC-STEMI study enrolled 448 STEMI patients, 217 of which were randomly allocated to the PPCI group and 231 to the RIC + PPCI group. The baseline characteristics and clinical presentation of both groups were previously published [11]. To highlight that a higher concentration of haemoglobin in the RIC + PPCI group (RIC + PPCI group 14.2 [13–15.4] g/dl vs PPCI group:13.9 [12.8–15] g/dL; *p* = 0.035) was the only statistical difference between groups.

Regarding the time of PPCI, no significant differences were detected. PPCI was performed in the afternoon period in 53% (n = 116) of the PPCI group and 50% (*n* = 116) of the RIC + PPCI group.

In this post-hoc analysis of the RIC-STEMI study, the study population was divided into 2 groups, according to the time of PPCI, and Table 1 shows baseline characteristics as well as clinical presentation of night-morning and afternoon groups.

**Table 1:** Baseline characteristics and clinical presentation of the night-morning group and the afternoon group

	Night-morning group (*n* = 216)	Afternoon group (*n* = 232)	*p *value
Baseline characteristics			
Age, years, median [IQ]	59 [50–70]	61 [52–72]	0.08
Female, *n* (%)	**34 (16)**	**55 (24)**	0.04*
BMI, kg/m^2^, median [IQ]	25 [24–27]	26 [24–29]	0.34
Systemic hypertension, *n* (%)	100 (46)	120 (52)	0.25
* Diabetes* Mellitus, *n* (%)	51 (24)	74 (32)	0.05
Hypercholesterolemia, *n* (%)	101 (47)	123 (53)	0.29
Smoker, *n* (%)	132 (61)	132 (57)	0.37
Previous IHD, *n* (%)	20 (9)	27 (12)	0.42
Previous PCI and/or CABG, *n* (%)	16 (7)	16 (7)	0.83
Medication			
Aspirin, *n* (%)	30 (14)	31 (13)	0.87
Second antiplatelet, *n* (%)	6 (3)	11 (5)	0.28
Statin, *n* (%)	55 (26)	76 (33)	0.09
ß-Blockers, *n* (%)	29 (13)	39 (17)	0.32
ACEi/ARB, *n* (%)	73 (34)	88 (38)	0.36
Nitrate, *n* (%)	5 (2)	6 (3)	0.85
Clinical presentation			
Systolic pressure, mmHg, median [IQ]	129 [110–149]	126 [110–141]	0.28
Admission ejection fraction, %, median [IQ]	45 [37–52]	45 [37–53]	0.73
Admission LVEF < 35%,* n* (%)	42 (19)	43 (19)	0.82
Creatinine, mg/dL, median [IQ]	0.9 [0.8–1.1]	0.9 [0.8–1.1]	0.90
Hemoglobin, g/dl, median [IQ]	14 [13–15]	14 [13–15]	0.47
48 h Troponin I level area under the curve, ng/mL, median [IQ]	43 [22–77]	38 [17–73]	0.12**
Killip scale at admission, *n* (%)			
I	185 (86)	202 (87)	0.25
II	23 (11)	27 (12)	
III	8 (4)	3 (1)	
TIMI 0 (occluded artery), *n* (%)	167 (77)	184 (79)	0.608
TIMI 1,* n* (%)	3 (1.4)	2 (0.9)	0.676
TIMI 2, *n* (%)	25 (11.6)	26 (11.2)	0.903
TIMI 3, *n* (%)	21 (9.7)	20 (8.6)	0.686
Anterior MI, *n* (%)	95 (44)	101 (44)	0.92
Ischemia–reperfusion time, hours, median [IQ]	3.8 [2.8–7.0]	3.6 [2.5–5.8]	0.27
Ischemia–reperfusion time > 3 h, *n* (%)	141 (66)	142 (62)	0.37
RIC as an adjuvant to PPCI, *n* (%)	115 (53)	116 (50)	0.50

Statistically significant values are in bold

*ACEi/ARB* angiotensin converter enzyme inhibitor/angiotensin II receptor blocker, *AUC* area under the curve; *BMI* body mass index, *CABG* coronary artery bypass grafting, *LVEF < 35%* Left ventricle ejection fraction, *PCI* percutaneous coronary intervention, MI−myocardial infarction, *RIC* remote ischemic conditioning

*p < 0.05

**Man−Whitney on the log−transformed AUC data

In addition to the balanced number of patients randomized to perform RIC, both groups had statistically similar characteristics, except for gender distribution, with a higher frequency of female gender in the afternoon group (24% vs 16%, *p* = 0.04).

During hospitalization, no divergence was found for 48-h troponin I level area under the curve (AUC) (trapezoid rule) between groups (Night-morning group: 43 [22–77] ng/mL vs afternoon group: 38 [17–73] ng/mL; *p* = 0.12). Regarding clinical presentation, anterior MI was reported in 44% of both groups, admission EF lower than 35% was observed in 19% of both groups and 77% of the night-morning group and in 79% of the afternoon group had occluded artery (TIMI 0) at the time of PPCI.

After confirming the homogeneity of groups, we evaluated the relation of the time of PPCI with follow-up results and no statistical difference was found (Table 2). There was no difference in follow-up EF (night-morning group: 54 [11] % vs afternoon group 55 [11] %, *p* = 0.26) between both groups.

**Table 2:** Relation of the time of PPCI with follow-up results

	Night-morning group (*n* = 216)	Afternoon group (*n* = 232)	*P* value	Effect size
Follow-up LVEF < 35%, *n* (%)	17 (10)	17 (9)	0.77	Φ = − 0.015
Follow-up LVEF, %, median [IQ]	54 [11]	55 [11]	0.26	Φ = − 0.051
Hospitalization due to HF, *n* (%)	18 (8)	12 (5)	0.18	Φ = − 0.063
Cardiac mortality, *n* (%)	8 (4)	8 (3)	0.89	Φ = − 0.007
All-cause mortality, *n* (%)	27 (13)	23 (10)	0.39	Φ = − 0.041
Primary follow-up endpoint (cardiac mortality + hospitalization due to HF), *n* (%)	21 (10)	13 (6)	0.1	Φ = − 0.078

*HF* heart failure, *LVEF < 35%* left ventricle ejection fraction, *MACCE* major adverse cardiovascular and cerebrovascular events on follow-up, *MI* myocardial infarction; *p < 0.05

Nevertheless, a survival analysis was performed using the Cox regression model by the forward method, to verify whether the afternoon period is an independent predictor of the primary follow-up endpoint.

Table 3 shows the regression model. The last stage of the model was statistically significant [*X*^2^ (6) = 85,688; *p* < 0,001] and revealed that the afternoon period was an independent predictor of the lower primary follow-up endpoint (HR = 0.474; 95% CI 0.230–0.977; *p* = 0.043). In addition, admission EF less than 35%, HF during hospitalization, 48 h Troponin I level AUC, admission creatinine and RIC were also independent predictors.

**Table 3:** Cox regression model for primary endpoint follow-up

Models	Variables	*p* value	HR	95.0% CI para HR	
				Inferior	Superior
Stage 1					
* X*^2^(1) = 26.633; *p* < 0.001*	48 h troponin I level AUC	< 0.001*	1.007	1.005	1.009
Stage 2					
* X*^2^(2) = 62.074; *p* < 0.001*	HF during hospitalization	< 0.001*	9.828	4.452	21.696
	48 h troponin I level AUC	< 0.001*	1.005	1.002	1.007
Stage 3					
* X*^2^(3) = 72.426; *p* < 0.001*	Admission EF < 35%	0.002*	3.560	1.607	7.885
	HF during hospitalization	< 0.001*	6.112	2.598	14.379
	48 h troponin I level AUC	0.001*	1.004	1.002	1.006
Stage 4					
* X*^2^(4) = 77.397; *p* < 0.001*	Admission EF < 35%	0.001*	4.065	1.833	9.017
	HF during hospitalization	< 0.001*	4.907	2.027	11.879
	48 h Troponin I level AUC	< 0.001*	1.004	1.002	1.006
	Creatinine	0.017*	2.312	1.159	4.612
Stage 5					
* X*^2^(5) = 81.423; *p* < 0.001*	Admission EF < 35%	< 0.001*	4.306	1.952	9.500
	HF during hospitalization	< 0.001*	4.858	2.022	11.672
	RIC	0.052*	0.469	0.218	1.007
	48 h Troponin I level AUC	0.005*	1.003	1.001	1.006
	Creatinine	0.022*	2.242	1.121	4.485
Stage 6					
* X*^2^(6) = 85.688; *p* < 0.001*	Admission EF < 35%	0.00*	4.181	1.900	9.196
	HF during hospitalization	0.001*	4.657	1.943	11.166
	**RIC**	0.029*	**0.423**	**0.195**	**0.917**
	**Afternoon period**	0.043*	**0.474**	**0.230**	**0.977**
	48 h Troponin I level AUC	0.003*	1.004	1.001	1.007
	Creatinine	0.022*	2.294	1.125	4.677

Bold highlights that the results were statistically significant

*AUC* area under the curve, *EF* ejection fraction; *HR* hazard ratio; *HF* heart failure; *RIC* remote ischemic conditioning *p<0.05

Finally, in this post-hoc analysis, there was a statistically lower frequency of hospitalization due to HF (afternoon group: 0.9% vs 9.5%, *p* = 0.003) and primary follow-up endpoint (afternoon group: 0.9% vs 10.3%, *p* = 0.002) in the RIC + PPCI group only in the afternoon period (Table 4).

**Table 4:** Comparasion of follow-up clinical results between the PPCI group and the RIC group according to the time of PPCI

	PPCI group (*n* = 217)	RIC group (*n* = 231)	*P* value	Effect size
Hospitalization due to HF, *n* (%)				
NM group	9 (9)	9 (8)	0.42	Φ = − 0.020
A group	**11 (9.5)**	**1 (0.9)**	0.003*	**Φ = **− **0.195**
MACCE, *n* (%)				
NM group	17 (17)	13 (11)	0.24	Φ = − 0.080
A group	16 (14)	9 (8)	0.14	Φ = − 0.097
MI, *n* (%)				
NM group	4 (4)	3 (3)	0.71	Φ = − 0.038
A group	5 (4)	4 (3)	1.00	Φ = − 0.022
Cardiac mortality, *n* (%)				
NM group	5 (5)	3 (3)	0.48	Φ = − 0.062
A group	7 (6)	1 (0.9)	0.07	Φ = − 0.142
All-cause mortality, *n* (%)				
NM group	15 (15)	12 (10)	0.33	Φ = − 0.067
A group	15 (13)	8 (7)	0.12	Φ = − 0.101
Primary follow-up endpoint (cardiac mortality + hospitalization due to HF), *n* (%)				
NM group	12 (12)	9 (8)	0.316	Φ = − 0.068
A group	**12 (10.3)**	**1 (0.9)**	0.002*	**Φ = **− **0.206**

Bold highlights that the results were statistically significant

*A group* afternoon group, *HF* heart failure, *NM* group−night−morning group, *MACCE* major adverse cardiovascular and cerebrovascular events on follow−up, *MI* myocardial infarction. *P < 0.05

Considering secondary follow-up endpoints, hospitalizations due to HF were also less frequent in the RIC + PPCI group only in the afternoon period (afternoon group: 0.9% vs 10%, *p* = 0.003).

There were no significant differences between PPCI and RIC + PPCI group, in both periods, regarding MACCE, MI, cardiac mortality and total mortality.

A survival analysis of the afternoon period (Table 5) was performed using the Cox regression model through the forward method and confirmed, in the regression model [*X*^2^(5) = 51,555; *p* < 0,001], that RIC remains a statistically significant independent predictor of primary follow-up endpoint (HR = 0.098; 95% CI 0.012–0.785; *p* = 0.029).

**Table 5:** Cox regression model for primary endpoint follow-up in the afternoon group

Models	Variables	*p* value	HR	95.0% CI para HR	
				Inferior	Superior
Stage 1					
*X*^2^(1) = 15.959; *p* < 0.001*	48 h Troponin I level AUC	< 0.001*	1.007	1.004	1.009
Stage 2					
*X*^2^(2) = 26.573; *p* < 0.001*	HF during hospitalization	0.001*	8.181	2.311	28.956
	48 h Troponin I level AUC	0.001*	1.005	1.002	1.007
Stage 3					
*X*^2^(3) = 31.477; *p* < 0.001*	Admission EF < 35%	0.032*	4.410	1.137	17.104
	HF during hospitalization	0.024*	4.776	1.230	18.540
	48 h Troponin I level AUC	0.009*	1.004	1.001	1.006
Stage 4					
*X*^2^(4) = 40.798; *p* < 0.001*	Admission EF < 35%	0.002*	11.122	2.472	50.046
	HF during hospitalization	0.276	2.185	0.535	8.923
	48 h Troponin I level AUC	0.003*	1.005	1.002	1.008
	Creatinine	0.002*	5.986	1.964	18.244
Stage 5					
*X*^2^(3) = 39.570; *p* < 0.001*	Admission EF < 35%	< 0.001*	14.313	3.455	59.295
	48 h Troponin I level AUC	< 0.001*	1.005	1.002	1.008
	Creatinine	< 0.001*	8.180	3.015	22.194
Stage 6					
*X*^2^(4) = 46.599; *p* < 0.001*	Admission EF < 35%	< 0.001*	13.471	3.316	54.717
	RIC	0.043*	0.119	0.015	0.934
	48 h Troponin I level AUC	0.002*	1.004	1.002	1.007
	Creatinine	** < 0.001**	8.189	2.959	22.667
Stage 7					
*X*^2^(5) = 51.555; *p* < 0.001*	Age	0.028*	1.057	1.006	1.110
	Admission EF < 35%	< 0.001*	15.868	3.817	65.966
	RIC	0.029*	0.098	0.012	0.785
	48 h Troponin I level AUC	0.013*	1.004	1.001	1.007
	Creatinine	**< 0.001**	9.981	3.062	32.534

Statistically significant values are in bold

AUC−area under the curve; EF−ejection fraction; HR−Hazard Ratio; HF heart failure; RIC−remote ischemic conditioning * P<0.05

However, in the survival analysis of the night-morning group (Table 6), RIC was not an independent predictor of the primary follow-up endpoint.

**Table 6:** Cox regression model for primary endpoint follow-up in the night-morning group

Models	Variables	p-value	HR	95.0% CI para HR	
				Inferior	Superior
Stage 1					
*X*^2^(1) = 30.086; *p* < 0.001*	HF during hospitalization	< 0.001*	12.624	4.605	34.608
Stage 2					
*X*^2^(2) = 36.798; *p* < 0.001*	HF during hospitalization	< 0.001*	7.459	2.466	22.565
	Admission EF < 35%	0.012*	3.462	1.312	9.135
Stage 3					
*X*^2^(3) = 31.477; *p* < 0.001*	Admission EF < 35%	0.023*	3.143	1.171	8.438
	HF during hospitalization	< 0.001*	6.384	2.071	19.679
	48 h Troponin I level AUC	0.037*	1.005	1.000	1.009

*AUC* area under the curve, *EF* ejection fraction, *HR*−Hazard Ratio, *HF* heart failure. *P < 0.05

**Fig. 2:**
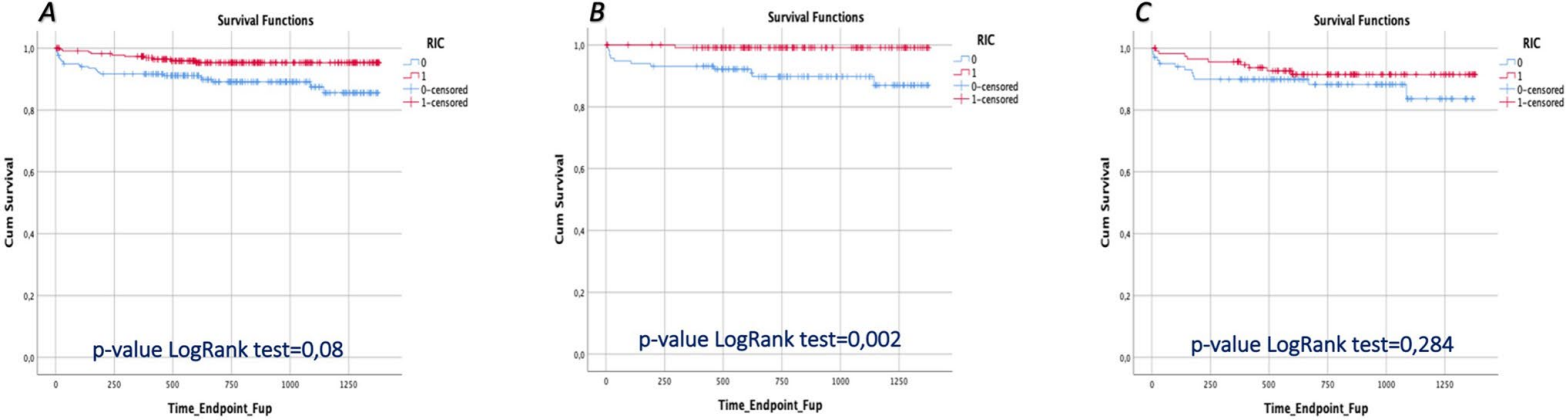
**A**–**C** Kaplan Meier curves: **A** RIC-STEMI population: impact of RIC on primary follow-up endpoint; **B** Afternoon group: impact of RIC on primary follow-up endpoint; **B**, **C** Night-morning group: impact of RIC on primary follow-up endpoint

These results were supported by the Kaplan Meier curves which showed that RIC had a significant impact on time without a primary follow-up endpoint in STEMI patients included in RIC-STEMI study (Fig. 2A) (*p *value LogRank test = 0,08) and in our afternoon group (Fig. 2B) (*p* value LogRank test = 0,002), but not in a night-morning group (Fig. 2C).

Finally, a survival analysis of the PPCI group was performed and the afternoon period was not an independent predictor of the primary follow-up endpoint (Table 7).

**Table 7:** Cox regression model for the primary endpoint in the PPCI group

Models	Variables	*p* value	HR	95.0% CI para HR	
				Inferior	Superior
Stage 1					
* X*^2^(1) = 19.903; *p* < 0.001*	48 h troponin I level AUC	< 0.001*	1.006	1.004	1.008
Stage 2					
* X*^2^(2) = 33.407; *p* < 0.001*	HF during hospitalization	< 0.001*	5.491	2.210	13.646
	48 h troponin I level AUC	< 0.001*	1.004	1.002	1.006
Stage 3					
* X*^2^(3) = 39.723; *p* < 0.001*	Admission EF < 35%	0.011*	3.390	1.316	8.730
	HF during hospitalization	0.005*	3.916	1.496	10.254
	48 h Troponin I level AUC	0.012*	1.003	1.001	1.006
Stage 4					
* X*^2^(4) = 45.007; *p* < 0.001*	Admission EF < 35%	0.005*	3.846	1.518	9.742
	HF during hospitalization	0.039*	2.907	1.054	8.020
	48 h Troponin I level AUC	0.004*	1.004	1.001	1.006
	Creatinine	0.014*	2.686	1.218	5.926

*AUC* area under the curve, *EF* ejection fraction, *HR* Hazard Ratio, *HF* heart failure *p < 0.05

## Discussion

In opposition to previous studies [8–11], Hausenloy et al. in CONDI2/ERICPPCI [12], the largest prospective multicenter study, did not reveal beneficial effects of RIC in reducing cardiac mortality or hospitalization due to HF. Despite its great value, this study had some particularities, which may have influenced the results. To point out: a short follow-up time (12 months), inadequate for the detection of HF events secondary to ventricular remodeling and a median ischemia time of less than 3 h. Considering that patients with anterior MI and a total ischemia time between 3 to 8 h benefited the most from RIC [16], the shorter time may have compromised the RIC impact. At the very least, these results reinforce an already known truth, that RIC is not effective under all circumstances [17]. Several factors influence the benefit of RIC, namely: age, history of ischemic coronary disease, total ischemia time, infarct size and localization, occluded artery at the time of RIC, comedication and comorbidity [16, 18]. There may be even other factors that have not been described yet, with a possible impact on the response to RIC.

This study is a post-hoc analysis of the RIC-STEMI study [11], a single-center randomized controlled trial. A previous pre-specified sub-analysis had already exposed that RIC only had a benefit in populations with anterior MI, total ischemia time greater than 3 h and occluded artery at the time of RIC. Our study had similar groups with well-balanced characteristics, except for a higher proportion of women in the afternoon group; however, no justification was discovered to explain this finding. No differences were found regarding age, anterior MI, total ischemia time and the proportion of occluded artery at the time of RIC. The analysis of the time-of-day reperfusion impact on the clinical results in STEMI patients revealed a much lower frequency of primary follow-up endpoint when RIC was performed as an adjuvant to PPCI in the afternoon. These results occurred without differences in 48 h troponin I levels AUC. Indeed, Gaspar et al. (2018) and Sloth et al. (2014) already failed to show reduced infarct size through troponin levels despite improvement in clinical results [8, 11]. Perhaps this biomarker is not the most accurate way to access infarct size, and an imagological evaluation should be preferred as in LIPSIA CONDITIONING STUDY [10]. Regarding the lack of differences in follow-up ejection fraction, this could be due to the fact that some participants died before doing the follow-up echocardiogram which could have had an impact on the study results.

A relation between MI and day-time variation was previously reported considering the higher incidence and larger dimensions of MI in the early morning [19–23]. These findings could be explained by the endogenous circadian fluctuation of thrombolytic activity, platelet aggregation and adrenergic activation, which some studies describe as being increased in the early morning [24–26]. In addition, Montaigne et al., also showed a relation between daytime variation and cardiomyocyte tolerance to IRI, higher in the afternoon, in patients who underwent aortic valve replacement surgery. This finding was concomitant with transcriptional alterations in the expression of the circadian gene of Rev-Erbα [14]. Furthermore, in two analyses of STEMI patients that underwent manual thrombus aspiration [21] and PPCI [27] smaller infarct size and better clinical results were found when symptoms began in the afternoon.

Our study points to the existence of a biorhythm for the cardioprotective effect of RIC since RIC was an independent predictor of the primary follow-up endpoint just in the afternoon group (HR = 0.098; 95% CI 0.012–0.785; *p* = 0.029). The afternoon period was not an independent predictor of the primary follow-up endpoint in the PPCI group.

Accordingly, our study revealed an important cardioprotective effect of RIC, namely in the afternoon period, suggesting that the afternoon period enhances the cardioprotection induced by RIC.

The authors consider that is still unclear whether RIC, restricted to high-risk patients, is cardioprotective and future clinical investigation should take into consideration the previously pointed features.

Therefore, larger studies are necessary to confirm these results and it would also be interesting to perform this sub-analysis in the studies previously published.

## Limitations

This study has limitations related to the fact that it was a post-hoc analysis of a single-center randomized controlled trial with a limited sample, which compromises the subgroup analysis. In this line, the number of events evaluated in the follow-up was also reduced, despite a mean follow-up of 2.1 years.

## Supplementary Information

Below is the link to the electronic supplementary material.

Supplementary file1 (PDF 63 kb)

## Data Availability

The datasets generated during and/or analysed during the current study are available from the corresponding author. All data analyzed in this study are included in this published article from Tables 1–7.

## Abbreviations

ACEi/ARB: Angiotensin converter enzyme Inhibitor/Angiotensin II receptor blocker
AUC: Area under curve
CABG: Coronary artery bypass grafting
CI: Confidence interval
CMR: Cardiac magnetic resonance
CRT: Cardiac resynchronization therapy device
EF: Ejection fraction
HF: Heart failure
HR: Hazard ratio
ICD: Cardioverter-defibrillator
IR: Interquartile range
IRI: Ischemia–reperfusion injury
MACCE: Major adverse cardiovascular and cerebrovascular events on follow-up
Mdn: Median
MI: Myocardial infarction
PCI: Percutaneous coronary intervention
PPCI: Primary percutaneous coronary intervention
RIC: Remote ischemic conditioning
STEMI: ST-elevation myocardial infarction

## References

[1] Townsend N, Nichols M, Scarborough P, Rayner M (2015). Cardiovascular disease in Europe-epidemiological update. Eur Hear J.

[2] De Luca G, Suryapranata H, Ottervanger JP, Antman EM (2004). Time delay to treatment and mortality in primary angioplasty for acute myocardial infarction: every minute of delay counts. Circulation.

[3] Heusch G, Gersh BJ (2017). The pathophysiology of acute myocardial infarction and strategies of protection beyond reperfusion: a continual challenge. Eur Hear J.

[4] Jernberg T, Johanson P, Held C, Svennblad B, Lindbäck J, Wallentin L (2011). Association between adoption of evidence-based treatment and survival for patients with ST-elevation myocardial infarction. JAMA - J Am Med Assoc.

[5] Yellon DM, Hausenloy DJ (2007). Myocardial reperfusion injury. N Engl J Med.

[6] Hausenloy DJ, Yellon DM (2013). Myocardial ischemia-reperfusion injury: a neglected therapeutic target. J Clin Invest.

[7] Chong J, Bulluck H, Yap EP, Ho AF, Boisvert WA, Hausenloy DJ (2018). Remote ischemic conditioning in ST-segment elevation myocardial infarction–an update. Cond Med.

[8] Sloth AD, Schmidt MR, Munk K, Kharbanda RK, Redington AN, Schmidt M, Pedersen L, Sorensen HT, BOTKER HE,  (2014). Improved long-term clinical outcomes in patients with ST-elevation myocardial infarction undergoing remote ischaemic conditioning as an adjunct to primary percutaneous coronary intervention. Eur Heart J.

[9] White SK, Frohlich GM, Sado DM, Maestrini V, Fontana M, Treibel TA, Tehrani S, Flett AS, Meier P, Ariti C, Davies JR, Moon JC, Yellon DM, Hausenloy H (2015). Remote ischemic conditioning reduces myocardial infarct size and edema in patients with ST-segment elevation myocardial infarction. JACC Cardiovasc Interv.

[10] Eitel I, Stiermaier T, Rommel KP, Fuernau G, Sandri M, Mangner N, Linke A, Erbs S, Lurz P, Boudriot E, Meinhard M, Desch SGA, Thiele H (2015). Cardioprotection by combined intrahospital remote ischaemic perconditioning and postconditioning in ST-elevation myocardial infarction: the randomized LIPSIA CONDITIONING trial. Eur Heart J.

[11] Gaspar A, Lourenço AP, Pereira MÁ, Azevedo P, Roncon-Albuquerque R, Marques J, Leite-Moreira AF (2018). Randomized controlled trial of remote ischaemic conditioning in ST-elevation myocardial infarction as adjuvant to primary angioplasty (RIC-STEMI). Basic Res Cardiol.

[12] Hausenloy DJ, Kharbanda RK, Møller UK, Ramlall M, Aarøe J, Butler R, Bulluck H, Clayton T, Dana A, Dodd M, Engstrom T, Evans R, Lassen JF, Christensen EF, Garcia-Ruiz JM, Gorog DA, Hjort J, Houghton RF, Ibanez B, Knight R, Lippert FK, Lønborg JT, Maeng M, Milasinovic D, More R, Nicholas JM, Jensen LO, Radovanovic N, Rakhit RD, Ravkilde J, Ryding AD, Schmidt MR, Riddervold IS, Sørensen HT, Stankovic G, Varma M, Webb I, Terkelsen CJ, Greenwood JP, Yellon DM, Bøtker HE, on behalf of theCONDI-2/ERIC-PPCI Investigators (2019). Effect of remote ischaemic conditioning on clinical outcomes in patients with acute myocardial infarction (CONDI-2/ERIC-PPCI): a single-blind randomised controlled trial. Lancet.

[13] Francis R, Chong J, Ramlall M, Bucciarelli-Ducci C, Clayton T, Dodd M, Engstrøm T, Evans R, Ferreira V, Fontana M, Greenwood JP, Kharbanda RK, Kim WY, Kotecha T, Lønborg JT, Mathur A, Møller UK, Moon J, Perkins A, Rakhit RD, Yellon DM, Bøtker HE, Bulluck H, Hausenloy DJ (2021). Effect of remote ischaemic conditioning on infarct size and remodelling in ST-segment elevation myocardial infarction patients: The CONDI-2/ERIC-PPCI CMR substudy. Basic Res Cardiol.

[14] Montaigne D, Marechal X, Modine T, Coisne A, Mouton S, Fayad G, Ninni S, Klein C, Ortmans S, Seunes C, Potelle C, Berthier A, Gheeraert C, Piveteau C, Deprez R, Eeckhoute J, Duez H, Lacroix D, Deprez B, Jegou B, Koussa M, Edme JL, Lefebvre P, Staels B (2018). Daytime variation of perioperative myocardial injury in cardiac surgery and its prevention by Rev-Erbα antagonism: a single-centre propensity-matched cohort study and a randomised study. Lancet.

[15] Ibanez B, James S, Agewall S, Antunes MJ, Bucciarelli-Ducci C, Bueno H, Caforio ALP, Crea P, Goudevenos JA, Halvorsen S, Hindricks G, Kastrati A, Lenzen MJ, Prescott E, Roffi M, Valgimigli M, Varenhorst C, Vranckx P, Widimský P (2018). 2017 ESC guidelines for the management of acute myocardial infarction in patients presenting with ST-segment elevation. Eur Heart J.

[16] Bell RM, Bøtker HE, Carr RD, Davidson SM, Downey JM, Dutka DP, Heusch G, Ibanez B, Macallister R, Stoppe C, Ovize M, Redington A, Walker JM, Yellon DM (2016). 9th Hatter Biannual Meeting: position document on ischaemia/reperfusion injury, conditioning and the ten commandments of cardioprotection. Basic Res Cardiol.

[17] Hausenloy D, Ntsekhe M, Yellon DM (2020). A future for remote ischaemic conditioning in high risk patients. Basic Res Cardiol.

[18] Ferdinandy P, Hausenloy DJ, Heusch G, Baxter GF, Schulz R (2014). Interaction of risk factors, comorbidities, and comedications with ischemia/reperfusion injury and cardioprotection by preconditioning, postconditioning, and remote conditioning. Pharmacol rev.

[19] Suárez-Barrientos A, López-Romero P, Vivas D, Castro-Ferreira F, Núñez-Gil I, Franco E, Núñez-Gil I, Franco E, Ruiz-Mateos B, García-Rubira JC, Fernández-Ortiz A, Macaya C, Ibanez B (2011). Circadian variations of infarct size in acute myocardial infarction. Heart.

[20] Fournier S, Eeckhout E, Mangiacapra F, Trana C, Lauriers N, Beggah AT, Monney P, Cook S, Bardy D, Vogt P, Muller O (2012). Circadian variations of ischemic burden among patients with myocardial infarction undergoing primary percutaneous coronary intervention. Am Heart J.

[21] Fournier S, Muller O, Benedetto U, Roffi M, Pilgrim T, Eberli FR, Rickli H, Radovanovic D, Erne P, Cook S, Noble S, Fesselet R, Zuffi A, Degrauwe S, Masci P, Windecker S, Eeckhout E, Iglesias JF (2018). Circadian dependence of manual thrombus aspiration benefit in patients with ST-segment elevation myocardial infarction undergoing primary percutaneous coronary intervention. Clin Res Cardiol.

[22] Reiter R, Swingen C, Moore L, Henry TD, Traverse JH (2012). Circadian dependence of infarct size and left ventricular function after ST elevation myocardial infarction. Circ Res.

[23] Bulluck H, Nicholas J, Crimi G, White SK, Ludman AJ, Pica S, Raineri C, Cabrera-Fuentes HA, Yellon D, Rodriguez-Palomares J, Garcia-Dorado D, Hausenloy DJ (2017). Circadian variation in acute myocardial infarct size assessed by cardiovascular magnetic resonance in reperfused STEMI patients. Int J Cardiol.

[24] Tofler GH, Brezinski D, Schafer AI, Czeisler CA, Ritherford JD (1987). Concurrent morning increase in platelet aggregability and the risk of myocardial infarction and sudden cardiac death. N Engl J Med.

[25] Petralito A, Mangiafico R, Gibiino S, Cuffari MF,  Fiore C (1982). Daily modifications of plasma fibrinogen platelets aggregation, Howell’s time, PTT, TT, and antithrombin II in normal subjects and in patients with vascular disease. Chronobiologia.

[26] Scheer FAJL, Michelson AD, Frelinger AL, Evoniuk H, Kelly EE, McCarthy M, Doamekpor LA, Barnard MR, Shea SA (2011). The human endogenous circadian system causes greatest platelet activation during the biological morning independent of behaviors. PLoS ONE.

[27] De Luca G, Suryapranata H, Ottervanger JP, Vant Hof AWJ, Hoorntje JCA, Gosselink ATM, Dambriknk JHE, Zijlstra F, Boer MJ (2005). Circadian variation in myocardial perfusion and mortality in patients with ST-segment elevation myocardial infarction treated by primary angioplasty. Am Heart J.

