# Supplementary material for: The impact of time-of-day reperfusion on remote ischemic conditioning in ST-elevation myocardial infarction: a RIC-STEMI substudy
Source: Heart Vessels. 2023 Mar 17;38(7):909–18. doi: 10.1007/s00380-023-02247-8 (PMC10209246; doi:10.1007/s00380-023-02247-8)
Supplement: Supplementary file 1 — Supplementary file1 (PDF 63 kb) [file 380_2023_2247_MOESM1_ESM.pdf]

Supplementary material

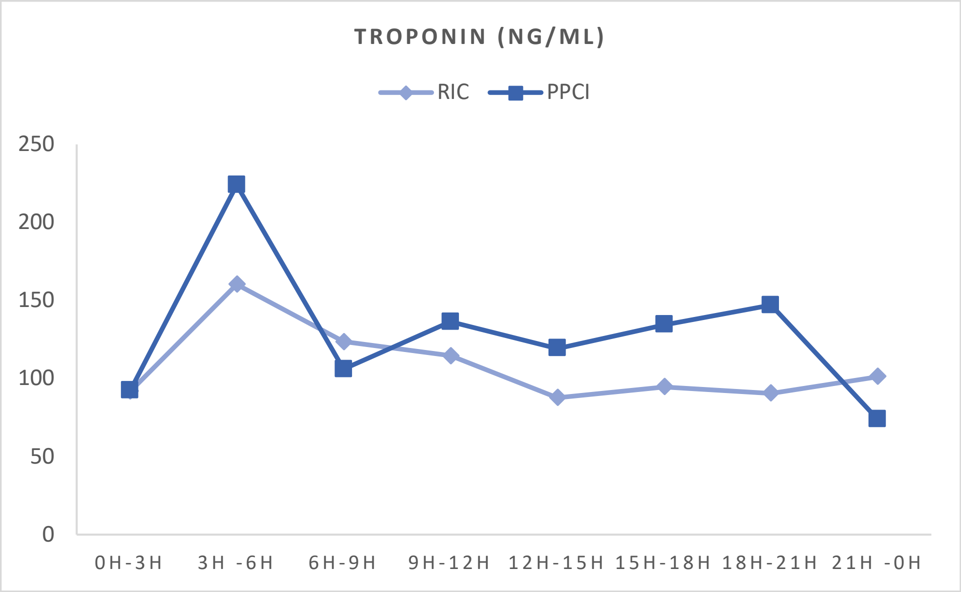

Graphic S1. 24-hour curves divided 3-hour intervals for mean troponin considering if the patient was included in PPCI group or RIC group.

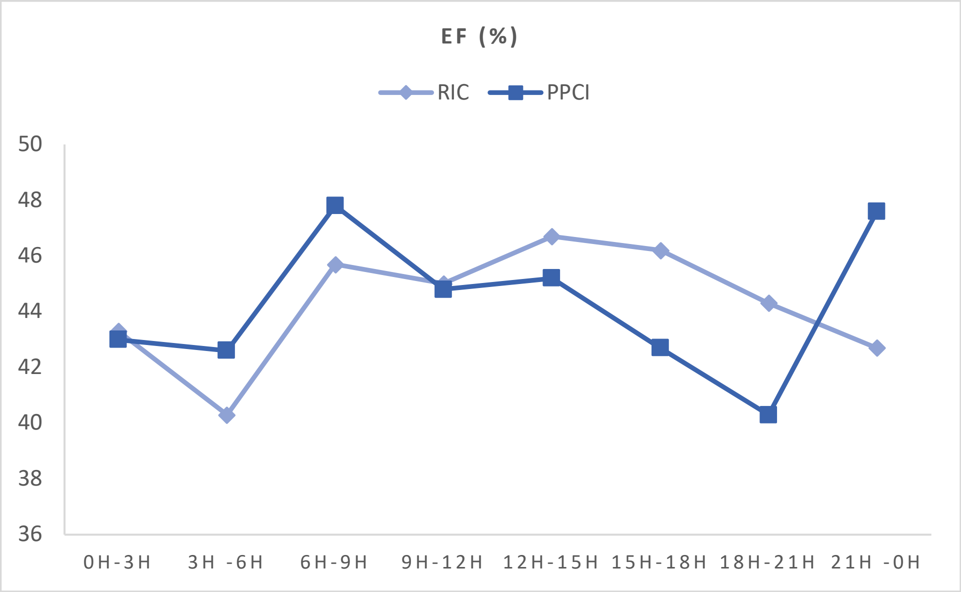

Graphic S2. 24-hour curves divided 3-hour intervals for admission ejection fraction considering if the patient was included in PPCI group or RIC group.
